# Caregiver descriptions of dystonia in cerebral palsy

**DOI:** 10.1002/acn3.51941

**Published:** 2024-01-04

**Authors:** Fayza Jaleel, Alyssa Rust, Shirley Cheung, Toni S. Pearson, Keisuke Ueda, Amy Robichaux‐Viehoever, Katie Leger, Keerthana Chintalapati, Danielle Guez‐Barber, Michele Shusterman, Bhooma Aravamuthan

**Affiliations:** ^1^ Division of Pediatric Neurology, Department of Neurology Washington University School of Medicine St. Louis Missouri USA; ^2^ Division of Neurology, Nationwide Children's Hospital Ohio State University Columbus Ohio USA; ^3^ Division of Child Neurology, Department of Pediatrics Children's Hospital of Philadelphia Philadelphia Pennsylvania USA; ^4^ Department of Neurology, Perelman School of Medicine University of Pennsylvania Philadelphia Pennsylvania USA; ^5^ The Cerebral Palsy Research Network Salt Lake City Utah USA

## Abstract

**Objective:**

To determine how caregivers describe dystonia in people with cerebral palsy (CP).

**Methods:**

In this prospective cohort study, paper surveys were administered to caregivers between September 7, 2021 and October 28, 2021 during CP Center visits at a large tertiary care center. Caregivers were asked to describe involuntary movements triggered by voluntary movement or triggered by tactile stimulation in the people with CP they cared for. Their CP Center medical provider separately assessed people with CP for dystonia. Movement features described exclusively by caregivers of people with CP and dystonia were determined using conventional content analysis.

**Results:**

113 caregivers responded on behalf of 56 people with and 57 people without dystonia. If caregivers noted that both voluntary movement and tactile stimulation triggered involuntary movements, that had a 92% positive predictive value for a dystonia diagnosis. Movement features exclusively described in people with CP and dystonia included: (1) stiffening, tensing, or tightening (15% of respondents); (2) involvement of the head (10%), torso (5%), or feet (5%); and (3) triggers of stretching (12.5%), excitement (5%), or transfers (5%).

**Interpretation:**

In addition to a thorough exam, asking caregivers of people with CP to describe involuntary movements triggered by voluntary movement or tactile stimulation may inform clinical dystonia diagnosis.

## Introduction

Dystonia in cerebral palsy (CP) is common and functionally debilitating, affecting at least 1 of every 1000 children in the US.^1^, ^2^ However, it often goes undiagnosed.^1^, ^3^, ^4^, ^5^, ^6^ For example, only 13% of people with CP and leg dystonia identified by expert consensus review of a neurologic exam video (the diagnostic gold standard) have that leg dystonia identified in a contemporaneous clinic visit with their CP medical provider.^6^ Therefore, a single clinical evaluation by even a well‐trained CP medical provider is insufficient to diagnose dystonia. This is a critical problem: dystonia in people with CP may be more likely to respond to treatment when recognized early.^7^

Currently, dystonia diagnosis is exclusively reliant on medical provider expertise and, ideally, on the diagnostic consensus of a group of experts.^8^ Tools to aid dystonia diagnosis in CP exist for the medical provider, most notably the Hypertonia Assessment Tool (HAT).^9^ However, even the HAT notes that dystonic movements may be “subtle” and may therefore be easy to miss.^9^, ^10^ Furthermore, the HAT and other scales meant to be utilized by medical providers require dystonia assessment during a discrete time period (e.g., a clinic visit).^9^, ^11^, ^12^, ^13^, ^14^ Not only do rates of dystonia diagnosis clinically improve with longitudinal assessment,^6^ but because dystonia is variable by definition, longitudinal knowledge of a person with CP may be inherently valuable for accurate dystonia diagnosis.^15^, ^16^

To improve access to prompt dystonia diagnosis and facilitate comprehensive assessment of dystonia outside of the clinic, dystonia diagnosis may be informed by caregiver expertise in addition to medical provider expertise. Recently, the Dyskinetic Cerebral Palsy Functional Impact Scale (D‐FIS) was developed as a tool that utilized caregiver responses to help gauge the functional impact of dystonia in people with CP.^11^ However, the D‐FIS requires that caregivers are already aware that the person with CP they care for has dystonia and are also aware of what that dystonia looks like.^11^ Scales or tools using caregiver expertise to facilitate dystonia *diagnosis* do not currently exist.

We assessed how caregivers of people with CP described potentially dystonic movements. We hypothesized that caregivers of people with CP and dystonia would describe their movements in a manner distinct from caregivers of people with CP without dystonia, and that these distinctions may be a useful aid for clinical dystonia diagnosis.

## Methods

This study was approved by the Washington University School of Medicine Institutional Review Board (ID# 202104140, 7/20/2021). Informed consent was obtained from participants after being given verbal and written descriptions of the study. Notably, the word “dystonia” was not used in the survey or consenting process.

### Respondents

Survey respondents were recruited during new and follow‐up clinic visits to the St. Louis Children's Hospital Cerebral Palsy Center between September 7, 2021 and October 28, 2021. The inclusion criterion was being a caregiver of a person with a CP diagnosis as confirmed by a CP Center medical provider per the 2006 consensus diagnostic criteria.^17^ The exclusion criterion was the caregiver not having fluency in English (noting that the survey was only provided in English).

### Survey development

To elicit movement descriptions that were most likely to represent descriptions of dystonia, survey questions were derived from the dystonia‐specific items of the HAT (Items 1, 2, and 6) which read as follows^9^:

Item 1. Increased involuntary movements/postures of the designated limb with the tactile stimulus of another body part.
Item 2. Increased involuntary movements/postures with purposeful movements of another body part.
Item 6. Increased tone with movement of another body part.

Of these items, Item 2 is thought to be the best performing and correlates with another dystonia assessment scale, the Barry Albright Dystonia Scale (BADS).^1^ Given this, HAT Item 2 was used to generate 1 question, and HAT Items 1 and 6 were combined to generate 1 question. These two survey questions were developed and iteratively refined by a pediatric movement disorders neurologist specializing in CP (BRA), an adult with CP and dystonia (FJ), a CP advocate and parent of a person with CP (MS), and a child neurologist and parent of a person with CP (DGB):

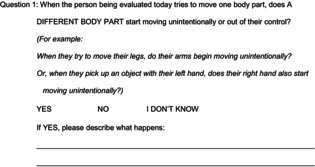

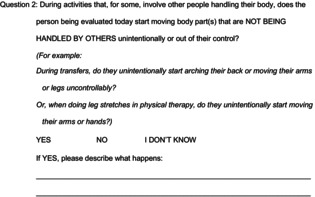

Surveys were distributed on paper at the beginning of the clinic visit and collected at the end of the clinic visit. The survey was completed by the caregiver independently without any input from medical providers.

### Dystonia diagnosis by the CP center medical provider

At the end of each visit, on the same day, the caregiver completed the survey, the CP Center medical provider was asked to write down the Gross Motor Function Classification System (GMFCS^18^) level of the person evaluated and whether or not the person had dystonia based on their clinical assessment (as informed by the history taken during the visit and their exam including observation of the person's movements with voluntary movement, tactile stimulation, and movement of another body part as prescribed by the HAT). Of note, the CP Center medical provider was not given any information about their caregiver's survey responses. CP Center medical providers were either pediatric neurologists who had completed pediatric movement disorders fellowship training or a dedicated pediatric neurology nurse practitioner specializing in cerebral palsy. We have previously established that rates of dystonia diagnosis do not differ between these types of providers in our center.^6^

### Qualitative analysis

Movement descriptions in response to the two survey questions were analyzed using a conventional content analysis approach. In the absence of an established framework for analyzing caregiver descriptions of movements in people with CP, we created and refined a novel codebook to characterize response content. Two investigators (FJ, AR) independently coded all responses. Discrepancies were resolved in consensus with adjudication by a third independent coder (BRA).

### Quantitative analysis

Rates of “yes,” “no,” and “I don't know” caregiver responses were tabulated to calculate sensitivity, specificity, positive predictive value (PPV), and negative predictive value (NPV) of these questions for medical provider‐identified dystonia. Frequencies of movement features uniquely described by caregivers of people with dystonia, but not unique to a single person, were tabulated (i.e., these movement features were described by at least two caregivers of people with CP and dystonia but not described by any caregivers of people with CP without dystonia).

### Statistical analysis

Demographic characteristics were compared between people with and without dystonia using Chi‐squared tests or *t*‐tests (with Welch's corrections for unequal standard deviations or Mann–Whitney tests for data that are not normally distributed as identified with Shapiro–Wilk testing), as appropriate. Binary logistic regressions were used to determine whether participant demographics were predictive of their responses to survey questions. The Chi‐square statistic and Wald test were used to determine the significance of the logistic regression model and model terms, respectively. Significance levels were set a priori at *p* < 0.05. Statistical analyses were performed using GraphPad Prism (version 8, GraphPad Software) and SPSS (version 28.0, IBM).

## Results

### Respondent demographics

**Figure 1:**
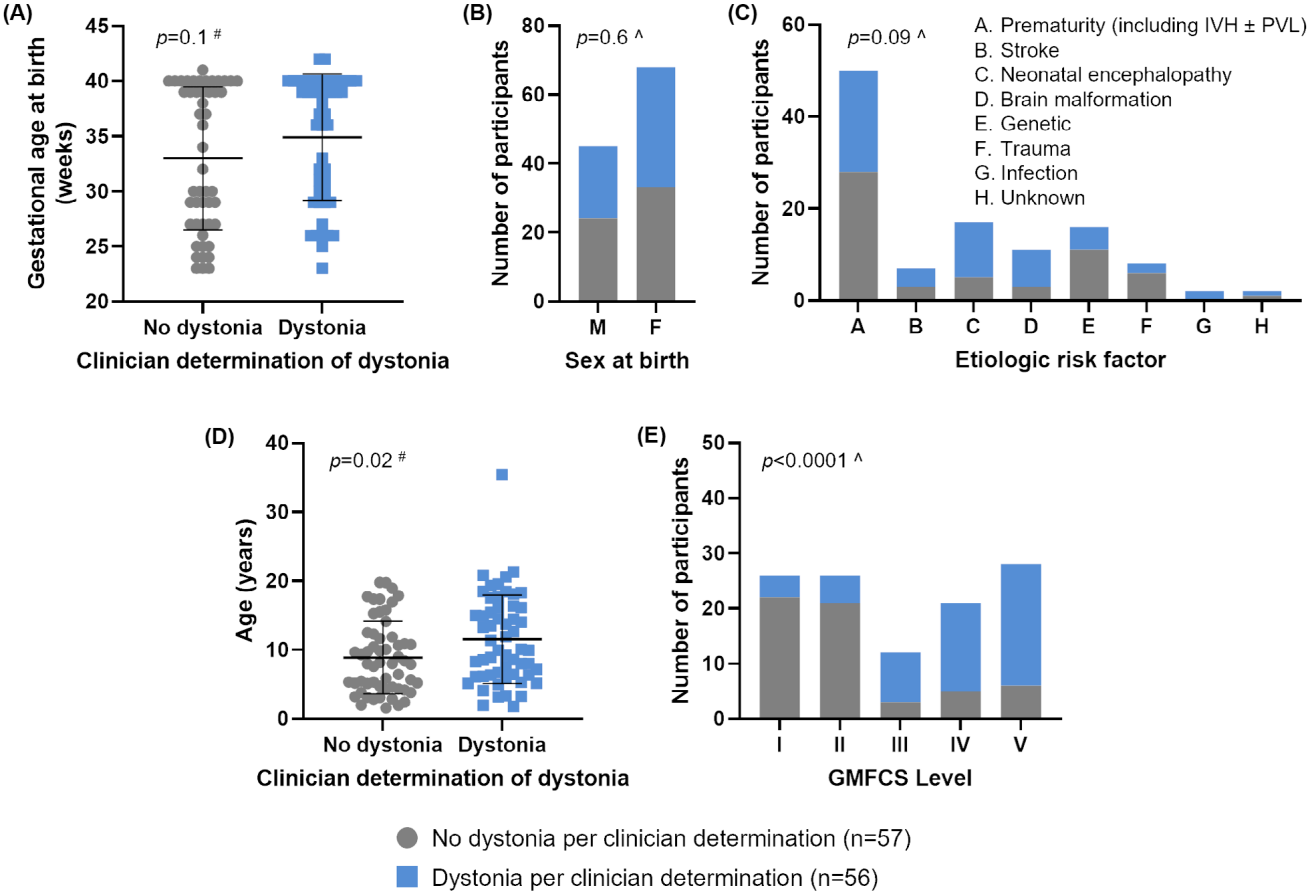
Participant demographics. Participant demographics are shown for those with and without dystonia (per clinician determination). Error bars in panels A and D show the mean ± standard deviation. M, male; F, female; PVL, periventricular leukomalacia associated with premature birth; IVH, intraventricular hemorrhage associated with premature birth; GMFCS, Gross Motor Function Classification System Level; #, *t*‐tests with Welch's corrections for unequal standard deviations or Mann–Whitney tests for data that are not normally distributed as identified with Shapiro–Wilk testing; ^, chi‐squared tests.

Two hundred people with CP were seen in the CP Center between September 7, 2021 and October 28, 2021. The caregivers of two people with CP were excluded because they were not fluent in English. Of the remaining 198 people with CP, caregivers of 113 people responded to this survey (57% response rate). There was no significant difference in sex, gestational age at birth, or distribution of CP etiologies between people with and without dystonia (Fig. 1A–C, Table S1). Of note, 56/113 people were identified as having dystonia by their medical provider (50%), in line with recent clinical and research‐based estimates of dystonia prevalence in people with CP.^1^, ^6^, ^8^, ^19^

People with dystonia in this study were significantly older (11 years [yr] 6 months [mo], 95% CI 9 yr 10 mo–13 yr 3 mo) than people without dystonia (8 yr 11 mo, 95% CI 7 yr 6 mo–10 yr 3 mo), though these age distributions were largely overlapping (Fig. 1D, Table S1). Of note, 13/113 (12%) people were over the age of 18, and 8/113 (7%) were under the age of 3.

Differences in the GMFCS level distribution between people with and without dystonia were more striking (*p* < 0.0001, Chi‐squared test) (Fig. 1E, Table S1). People at GMFCS levels I–III are independently ambulatory while those at GMFCS levels IV–V require a wheelchair for mobility.^18^ 68% (38/56) of people with dystonia were at GMFCS IV–V while only 19% (11/57) of people without dystonia were at GMFCS IV–V. Viewed another way, the majority of people at GMFCS IV–V were identified by medical providers as having dystonia (78%, 38/49) while only 28% (18/64) of people at GMFCS I–III were identified as having dystonia.

### Sensitivity, specificity, PPV, and NPV for dystonia in CP

Overall, “yes” responses to both questions had a 92% PPV for dystonia, with 96% specificity and 41% sensitivity for a dystonia diagnosis (Table 1). “No” or “I don't know” responses to both questions had a 77% NPV, with 75% specificity and 81% sensitivity for not having dystonia (Table 3).

**Table 1:** Sensitivity, specificity, and positive predictive value of caregiver responses for the presence of dystonia.

“Yes” responses	Overall			GMFCS I–III			GMFCS IV–V		
	Sensitivity (%)	Specificity (%)	PPV (%)	Sensitivity (%)	Specificity (%)	PPV (%)	Sensitivity (%)	Specificity (%)	PPV (%)
Q1 and Q2	41	96	92	39	98	88	42	91	94
Q1 or Q2	75	81	79	78	83	64	74	73	90
Q1	59	84	79	67	85	63	55	82	91
Q2	57	93	89	50	96	82	61	82	92

GMFCS, Gross Motor Function Classification System Level; GMFCS I–III, independently ambulatory; GMFCS IV–V, require a wheelchair for mobility; PPV, positive predictive value; Q1, “Yes” response to Survey Question 1; Q2, “Yes” response to Survey Question 2.

Although both age and GMFCS level distributions significantly differed between people with and without dystonia, binary logistic regression models including both of these variables revealed that only GMFCS level was a significant predictor of caregiver survey responses, but only to question 2 (regarding tactile triggers, Wald statistic 18.7, *p* < 0.001). Neither age nor GMFCS level were significant predictors of responses to question 1 (regarding voluntary movement triggers) (Table S2).

Given that GMFCS level was found to be a significant predictor of caregiver responses to at least one of the two survey questions, diagnostic metrics were compared between people at GMFCS I–III (independently ambulatory) vs. those at GMFCS IV–V (not independently ambulatory). The survey performed best for people at GMFCS I–III (88% PPV for “Yes” responses to both questions, 90% NPV for “No” or “I don't know” responses to both questions). Though the PPV of “Yes” responses to both questions remained high (94%) for people at GMFCS IV–V, the NPV of “No” or “I don't know” responses to both questions dropped to 44% (Tables 1 and 2).

**Table 2:** Sensitivity, specificity, and negative predictive value of caregiver responses for the absence of dystonia.

“No” or “I don't know” responses	Overall			GMFCS I–III			GMFCS IV–V		
	Sensitivity (%)	Specificity (%)	NPV (%)	Sensitivity (%)	Specificity (%)	NPV (%)	Sensitivity (%)	Specificity (%)	NPV (%)
Q1 and Q2	81	75	77	83	78	90	73	74	44
Q1 or Q2	96	41	63	98	39	80	91	42	31
Q1	84	59	68	85	67	87	82	55	35
Q2	93	57	69	96	50	83	82	61	38

GMFCS, Gross Motor Function Classification System Level; GMFCS I–III, independently ambulatory; GMFCS IV–V, require a wheelchair for mobility; NPV, negative predictive value; Q1, “No” or “I don't know” response to Survey Question 1; Q2, “No” or “I don't know” response to Survey Question 2.

### Movement features described exclusively by caregivers of people with CP and dystonia

**Figure 2:**
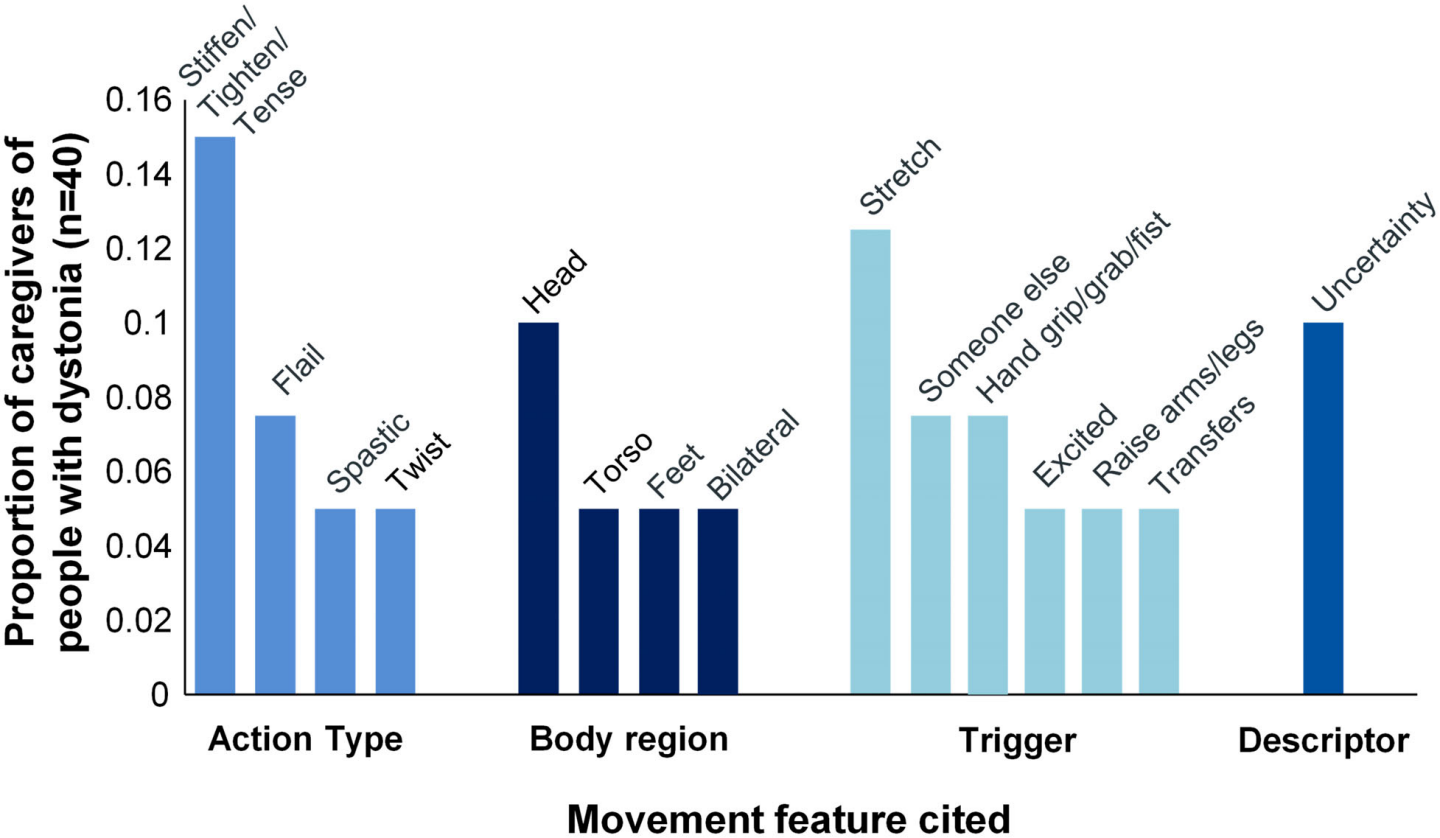
Movement features cited exclusively by caregivers of people with CP and dystonia.

Caregivers were asked to describe the relevant triggered involuntary movements that prompted their “Yes” responses to either survey question. Fifty caregivers (40 caregivers of people with dystonia, 10 caregivers of people without dystonia) provided such descriptions. Salient statements from these descriptions (called codes) were thematically grouped into five categories: action type, action body region, action descriptors, action triggers, and action alleviators (example codes and quotes in Table 3). Caregivers of people with dystonia were the only ones to describe 15 movement features across four code categories (Fig. 2).

**Table 3:** Abbreviated codebook.

Code category with code examples	Example caregiver movement descriptions
Action type	
Stiffen/tighten/tense	“other limbs stiffen” “right arm will tense up when working left” “her right arm/leg tightens”
Flail	“arms will stretch out and flail”
Spasticity	“sometimes it's just increased spasticity”
Twist	“torso twists to right”
Action body region	
Head	“when starts to speak, head moves + some torso twisting”
Torso	
Feet	“her feet might turn inward”
Bilateral	“when trying to use one arm both will move back”
Action descriptor	
Uncertainty	“it's hard to tell if it's unintentionally or not, but my guess is yes”
Variable	“sometimes when he uses one hand the other moves” “depends on what the person is doing”
Triggers	
Transfers	“tightening to right side during transfers, occasionally as well during stretches”
Stretches	
Someone else	“It often feels like she is working against you as you try to move or calm her”
Excitement	“gets excited and arms go up”
Raise arm(s)/leg(s)	“try to raise arms and leg will move” “when lifting one leg, other leg comes up”
Hand grip/grab/fist	“when grabbing an object with 1 hand, the other hand clenches into a fist” “sometimes when [Name's] arms/fist tighten, his legs will tighten” “gripping w/ left = right moves too; when he moves left hand, right hand seems to move as well, specially fingers”
Alleviators	
Stretch	“try stretch or push point behind knee or arch of foot to release area in other spot”
Sensory stimulus	

Underlined words indicate the specific parts of each caregiver description that are associated with each code.

The most common action type cited exclusively by caregivers of people with dystonia was stiffening/tightening/tensing (cited by 15%, *n* = 6/40). Although questions were designed to elicit descriptions of potential dystonia, only caregivers of people with dystonia described these involuntary movements with the terms “spastic” or “spasticity” (5%, 2/40).

Involuntary movements were described across multiple body regions, mainly in the arms or legs. However, only involuntary movements involving the head (10%, 4/40), torso (5%, 2/40), or feet (5%, 2/40) were exclusively cited by caregivers of people with dystonia. Both caregivers who described torso movements described “torso twisting” or “torso twists.” Only caregivers of people with dystonia noted that these triggered involuntary movements occurred bilaterally (5%, 2/40).

Six triggers of involuntary movements were described exclusively by caregivers of people with dystonia. Four of these were given as example triggers in the stems of the survey questions: stretch (cited by 12.5%, 5/40), someone else handling the person with CP (7.5%, 3/40), hand gripping/grabbing/fisting (7.5%, 3/40), and transfers (5%, 2/40). The remaining two triggers were cited without any possible priming from the question stems: excitement (5%, 2/40) and raising an arm/leg (5%, 2/40).

Only caregivers of people with dystonia described a sense of uncertainty about their movement descriptions (4%, 4/40), perhaps highlighting the difficult nature of dystonia identification even for people who have the greatest exposure to observing it. Finally, even though variability is a defining dystonia feature^15^, ^16^ often used by medical experts for leg dystonia assessment,^8^, ^20^ movement variability was cited at comparable frequencies by caregivers of people with dystonia (22.5%, 9/40) and caregivers of people without dystonia (30%, 3/10).

## Discussion

Though dystonia has been conceptualized as a diagnosis reliant exclusively on physical exam findings and medical expert consensus, our results suggest that history taking from caregivers likely provides useful information to inform dystonia diagnosis. If a caregiver noted that both voluntary movements and tactile stimulation triggered involuntary movements in the person with CP they cared for, those responses had a 92% PPV for medical provider‐identified dystonia. If caregivers noted that neither stimulus triggered involuntary movements, those responses had a 77% NPV for dystonia. These questions appeared to perform best for the people with CP at GMFCS I–III (88% PPV, 90% NPV), people for whom dystonia may be more frequently missed and may be more likely candidates for surgeries like selective dorsal rhizotomy for which dystonia is a relative contraindication.^21^ We also identified movement features exclusively cited by caregivers of people with dystonia, which may provide further diagnostic clues to the medical provider. In sum, these results advocate for the inclusion of caregivers as valuable participants in the dystonia diagnostic process.

### Implications for clinical practice

These results may be immediately valuable for clinical practice. Asking caregivers of people with CP about tactile or voluntary movement triggers for involuntary movements with the provision of some examples (as described in our two question survey) may be a useful aid for dystonia diagnosis. Descriptions of increased spasticity in response to the above triggers should not be automatically discounted; these descriptions may still be representative of dystonia or, at least, be co‐incident with dystonia. Our results also suggest that descriptions of movement or tone variability in response to these questions may not be a valuable differentiator between the presence or the absence of dystonia.

Caregiver responses to these questions are meant to inform dystonia diagnosis, but not replace the important role of the medical provider's assessment. It may be difficult to use caregiver responses to these questions alone to determine whether caregivers are describing dystonia or dystonia mimics (e.g., mirror movements). However, affirmative responses to both questions should prompt increased vigilance for dystonia in the clinic visit and can also be the beginning of a conversation with the family regarding how the movements they have described may affect the function of the person with CP.

### Implications for caregiver and family awareness of dystonia

A research priority shared by people with CP, caregivers, advocates, researchers, and clinicians is the development of methodologies to improve family awareness of dystonia in people with CP.^22^ Most people with CP in the US do not have easy access to tertiary care CP Centers or pediatric motor phenotyping experts. Providing broad access to the caregiver‐facing questions described here can help all medical practitioners identify people with CP who should be referred for formal evaluation for dystonia or, at the very least, should prompt conversations about the family's awareness of dystonia.

### High vigilance for dystonia in people with CP at GMFCS IV–V

We and others have demonstrated high rates of dystonia in people at GMFCS IV–V.^3^, ^4^ Though we are encouraged by the potential clinical utility of a caregiver‐facing two question assessment, the data from this study population suggests that identifying that a person with CP is at GMFCS levels IV–V, in and of itself, has moderate sensitivity (69%), specificity (81%), and PPV (78%) for dystonia. The vigilance for dystonia should be high when evaluating all people with CP, but this is particularly true for people with the greatest degrees of gross motor functional impairments: more likely than not, they have dystonia.

### The value of the “I don't know” response option

It is of note that caregiver‐driven response items were part of the initial development of the HAT but were removed from the final version “because caregivers and children had difficulty understanding and answering the questions posed.”^9^ This could also be a factor in our study, which we tried to address *a priori* by providing involuntary movement examples for the two survey questions we developed.

We additionally hypothesize that the presence of an “I don't know” response option for the two survey questions allowed caregivers to feel comfortable stratifying their answers by level of certainty. This is particularly notable because the only people who described uncertainty in their movement descriptions were caregivers of people with dystonia. Therefore, we would advise medical providers to explicitly allow caregivers to be unsure about how they describe the movements of the people with CP they care for. Descriptions of uncertainty may serve as a diagnostic clue.

### Limitations

The people we assessed were all followed in a tertiary care multidisciplinary CP center staffed by people with pediatric movement disorders and CP expertise. It is possible that these providers may have primed caregivers to describe movements in a certain way. We also did not assess how many caregivers were aware of the term “dystonia” or knew that the person with CP they cared for had dystonia. In addition to repeating this study over a longer time period and on a larger scale across multiple centers, it would be valuable to repeat this study for a population outside of a multidisciplinary CP Center and for caregivers who are not familiar with dystonia. This is particularly true for the qualitative portion of the analysis, which should be validated in a larger population.

It is notable that dystonia underdiagnosis is common, even amongst expert assessors.^6^ The fact that expert clinical assessment may be an imperfect gold standard makes this work all the more valuable. That is, anything that can trigger explicit consideration of dystonia in a clinic visit, including a caregiver‐facing survey, may have the potential to improve rates of dystonia diagnosis. Future work should test this hypothesis.

Co‐existing motor findings, like mirror movements, chorea, athetosis, ataxia, hypotonia, or spasticity may affect how caregivers describe dystonia and may also affect how medical providers assess dystonia. Though these potential contributions were not assessed here, it is notable that caregivers used terms like “spasticity” to describe their movements. The impact of co‐existing motor findings and treatments should be assessed in future work.

A key limitation of this study is the lack of information directly from people with CP. It is very likely that people with CP experience their own movements in a very different way from how others describe these movements. Repeating this study for adults with CP will be important.

### Conclusions

Caregivers should be asked to describe whether the person with CP they care for has involuntary movements triggered by voluntary movement or tactile stimulation. The responses to these questions may inform dystonia diagnosis and spark valuable discussions about the functional relevance of the potentially dystonic movements caregivers describe. These results can inform further studies to derive a more detailed caregiver‐driven dystonia diagnostic tool to facilitate comprehensive clinical dystonia assessment.

## Author Contributions

Fayza Jaleel helped design the study, carried out data analyses, and critically reviewed and revised the manuscript. Alyssa Rust carried out data analyses and critically reviewed and revised the manuscript. Shirley Cheung and Keerthana Chintalapati coordinated data collection and critically reviewed and revised the manuscript. Toni S. Pearson, Keisuke Ueda, Amy Robichaux‐Viehoever, and Katie Leger contributed to data collection and critically reviewed and revised the manuscript. Michele Shusterman and Danielle Guez‐Barber helped design the study, contributed to data interpretation, and critically reviewed and revised the manuscript. Bhooma Aravamuthan conceptualized and designed the study, supervised data collection and analysis, drafted the initial manuscript, and critically reviewed and revised the manuscript.

## Funding Information

NINDS 1K08NS117850‐01A1 (BRA).

## Conflict of Interest

The authors report no disclosures or conflicts of interest concerning the research related to this manuscript.

## Supporting information


**Table S1** Subject demographics.
**Table S2** Binary logistic regression results examining age and GMFCS levels as predictors of caregiver survey responses.

## Data Availability

All de‐identified data will be made available to qualified investigators upon request.
