# Supplementary material for: Caregiver descriptions of dystonia in cerebral palsy
Source: Ann Clin Transl Neurol. 2024 Jan 4;11(2):242–50. doi: 10.1002/acn3.51941 (PMC10863918; doi:10.1002/acn3.51941)
Supplement: Supplementary file 1 — Table S1 Subject demographics. Table S2 Binary logistic regression results examining age and GMFCS levels as predictors of caregiver survey responses. [file ACN3-11-242-s001.docx]

**Supplementary Table 1.** Subject demographics

| Demographic category | Value (mean with 95% CI or N with % of total) | |
| --- | --- | --- |
|  | No dystonia  (N=57) | Dystonia  (N=56) |
| Gestational age at birth, (mean weeks, 95% CI) | 33.0 (31.3-34.7) | 34.9 (33.4-36.4) |
| Sex (N, % male) | 24 (42%) | 12 (38%) |
| Etiologic risk factor  (N, % of total) |  |  |
| Prematurity | 28 (49%) | 22 (39%) |
| Stroke | 3 (5%) | 4 (7%) |
| Neonatal encephalopathy | 5 (9%) | 12 (21%) |
| Brain malformation | 3 (5%) | 8 (14%) |
| Genetic | 11 (19%) | 5 (9%) |
| Trauma | 6 (11%) | 2 (4%) |
| Infection | 0 (0%) | 2 (4%) |
| Unknown | 1 (2%) | 1 (2%) |
| Age (mean years, 95% CI) | 8.9 (7.5-10.2) | 11.5 (9.9-13.2) |
| GMFCS |  |  |
| I | 22 (39%) | 4 (7%) |
| II | 21 (37%) | 5 (9%) |
| III | 3 (5%) | 9 (16%) |
| IV | 5 (9%) | 16 (29%) |
| V | 6 (11%) | 22 (39%) |

**Supplementary Table 2.** Binary logistic regression results examining age and GMFCS levels as predictors of caregiver survey responses

| Responses | Overall Model Fit | | Age | | GMFCS | |
| --- | --- | --- | --- | --- | --- | --- |
|  | Chi-square statistic | *p* | Wald statistic | *p* | Wald statistic | *p* |
| Q1 | 11.3 | .047 | 1.9 | 0.2 | 8.1 | 0.09 |
| Q2 | 30.7 | <0.001 | 0.3 | 0.6 | 18.7 | <0.001 |

GMFCS – Gross Motor Function Classification System Level.
